# Palliative care needs of HIV exposed and infected children admitted to the inpatient paediatric unit in Uganda

**DOI:** 10.3332/ecancer.2014.489

**Published:** 2014-12-11

**Authors:** Jane Nakawesi, Ivy Kasirye, David Kavuma, Benjamin Muziru, Alice Businge, Jackie Naluwooza, Grace Kabunga, Yvonne Karamagi, Edith Akankwasa, Mary Odiit, Barbara Mukasa

**Affiliations:** Mildmay Uganda, PO Box 24985, Kampala, Uganda

**Keywords:** paediatric palliative care, HIV infected and exposed children, paediatric palliative care needs

## Abstract

Paediatric palliative care is an emerging subspecialty that focuses on achieving the best possible quality of life for children with life-limiting conditions and also for their families. It is a response to the suffering and unique needs of such children. Globally there is limited documented data available on the palliative care needs of children with HIV. A retrospective review of data of all the HIV exposed and positive children who were admitted to the ward from January to December 2012 was done to document their palliative care needs.

A total of 243 children were admitted to the ward during the stated period. Of these, 139 (57.2%) were female and 104 (42.8%) were male. Among them 131 (54%) were aged five years and below whereas 112 (46%) were above five years. Some of the identified palliative care needs documented included physical needs: pneumonia 46 (19%), severe acute malnutrition 38 (16%), mild and moderate acute malnutrition 23 (9.6%), and respiratory tract infections 22 (9.3%). Social needs: poor social support 21 (41%), financial instability 16 (31%), and child neglect 4 (8%). Psychological needs: antiretroviral treatment (ART) counselling 127 (36%), HIV counselling and testing for the child and family 63 (18%), adherence support 53 (15%), and others 11 (3%). Spiritual needs: discontinuing ART because of belief in spiritual healing 18 (81%), loss of hope because of severe ill health 1 (5%), and others 3 (14%).

These results emphasise the need for palliative care in children with HIV even in the era of ART. The needs identified are in keeping with studies done elsewhere and are similar to the palliative care needs of children with other life-limiting illnesses such as cancer.

**Conclusion:**

HIV positive and exposed children plus their families have vast palliative care needs and a holistic approach is the key in their management.

## Introduction

Paediatric palliative care is an emerging subspecialty that focuses on achieving the best possible quality of life for children with life-limiting conditions and also for their families. It is a response to the suffering and unique needs of such children [1]. Children with HIV continue to experience persistent treatment gaps [2]. In 2012, there were 647,000 children under 15 years receiving antiretroviral therapy (ART) globally. However, HIV treatment coverage for children (34%) remained half of coverage for adults (64%). Although the number of children receiving ART in 2012 increased by 14% in comparison to 2011, the pace of scale up was substantially slower than for adults (21%). The failure to expand access in many settings is because of lack of early infant diagnosis. This is an important reason why HIV treatment coverage remains much lower for children than for adults [2]. In Uganda only 39% of children born to HIV infected mothers are being tested within eight weeks of life. There is a very poor linkage between Prevention of Mother to Child Transmission of HIV (PMTCT) and Early Infant Diagnosis (EID) despite the high coverage of PMTCT achieved. In June 2013 the number of children in ART was 41,520, which is only 41% of those in need of it [3]. According to World Health Organisation (WHO) [26] palliative care is an essential component of a comprehensive package of care for people living with HIV and AIDS because of the variety of symptoms they can experience, such as pain, diarrhoea, cough, shortness of breath, nausea, weakness, fatigue, fever, and confusion. HIV and cancer are some of the major contributors to the palliative care burden for children in Africa [4]. A study of childhood cancers in Zambia found a significant rise in the paediatric malignancies because of the HIV epidemic [5]. There is, however,a limited data on the burden of cancer in Africa as well [6]. A study reviewing global cancer registration literature 2006–2008 found that less than 1% of the abstracts originated from Africa. Globally there is limited documented data available on the palliative care needs of children with HIV. This article reports on the palliative care needs of HIV exposed and HIV positive children admitted to the inpatient paediatric unit at Mildmay Uganda.

## Methods

### Study design

The study was a retrospective descriptive study of all HIV exposed and HIV positive children admitted to the inpatient unit at Mildmay Uganda from January to December 2012.

### Study setting—the inpatient unit at Mildmay Uganda

Mildmay Uganda is also a locally registered Non Governmental Organisation (NGO) and has three core functions: the provision of HIV prevention, care and treatment services at the hospital, training, education, and the provision of technical assistance to 16 districts in central Uganda for health system strengthening. Care and treatment are provided predominantly on an outpatient basis for most clients. In addition to this, there is a 33 bed capacity children’s ward, which provides care for critically and terminally ill children aged between 0–18 years. The hospital provides care and support to over 61,000 men, women, and children living with HIV at the main site and the districts, through a wide range of services. Of these, around 12% are babies and children below the age of 15.

Mildmay Uganda is one of the biggest single providers of ART in Uganda and currently has over 3500 children below the age of 15 on treatment.

Mildmay Uganda additionally provides high-quality training for health professionals and community volunteers. Training includes formal courses, clinical placements, and mentorships. This has had a substantial impact in improving paediatric care in Uganda, particularly in rural areas. The children’s inpatient unit provides specialist, acute, high-quality care for the most complicated cases of HIV in children. It is the only place of its kind in East Africa. A family-centred palliative care approach is used when delivering services in the inpatient unit. This holistic approach involves a multidisplinary team of clinicians, nurses, nutritionists, a physiotherapist, a play therapist, occupational therapists, counsellors, social workers, and pastoral care providers as recommended by WHO and other studies [27, 28]. Care for each child can therefore be highly personalised to meet their particular needs. Support is also provided for families through this multidisciplinary team. Children who come to the hospital are very sick as a result of their HIV—especially children who have been recently diagnosed with HIV and are not on medication, or those with aggressive diseases or terminal illnesses. They suffer from conditions such as severe malnutrition, malaria, pneumonia, tuberculosis, meningitis, or septicaemia. The services provided on the unit enable most of the babies and children to become well enough to take antiretroviral drugs which will prolong and improve their quality of life.

### Study participants

All HIV exposed and infected children admitted to the inpatient unit from January to December 2012.

### Data collection and analysis

Data were abstracted from the Mildmay Uganda patient electronic database (CAREWare) into Excel and then exported to Access 2007 for analysis. The variables abstracted for all the patients admitted to the inpatient unit from January to December 2012 included age, sex, HIV status, ART status, i.e., whether on ART or not, duration on ART for those on ART, duration of care at Mildmay Uganda, and the final diagnosis at admission. The psychosocial and spiritual needs were extracted from the social worker, counsellor, and spiritual counsellors who documented visits by the clients. The data extraction was done by the principal investigator. For the physical needs, the need was considered to be the final diagnosis. For the psychosocial and spiritual needs, it was taken as that reason for which the child and family had sought care or were referred to the specific member of the multidisciplinary team and any other need identified after assessment by this team member. This data was collected and analysed in Excel.

The analysis was purely descriptive. The descriptions included HIV status per age group, ART status per age group and gender, and duration on ART per age group and gender, duration of admission, proportions of children who presented with needs in the different categories i.e. physical, social, psychological, and spiritual.

### Ethical approval

No ethical approval was sought as this was more of an audit or review of data with no intervention to the patients. The data was obtained from the electronic database generated from their normal clinical management. However, Mildmay Uganda has a client consent form which patients and carers for children sign after informed consent. It is signed the day that the patient commences care and treatment at the hospital. This form gives permission to the hospital to use patients’ records and specimens for purposes of research, studies, and evaluations.

## Results

A total of 243 children were admitted to the ward during the stated period. Of these, 139 (57.2%) were female and 104 (42.8%) male. Among them, 131 (54%) were aged five years and below whereas 112 (46%) were above five years. A total of 200 (82%) had a confirmed HIV diagnosis whereas 43 (18%) did not have a confirmed HIV diagnosis so they were classified as HIV exposed. Regarding duration for which children had had HIV and treatment at Mildmay Uganda, the majority had been in care for more than five years, 112 (46%), whereas the others in care for a period of one to five years were 80 (33%), and those less than one year were 51 (21%) respectively. Of all these, 139 (57%) were on antiretroviral therapy whereas 104 (43%) were ART naïve. The majority of those on ART, 69 (69%) had been on it for more than five years. The majority of the patients were admitted for less than one week 107 (45%), and others for one to two weeks 70 (30%), two to four weeks 46 (19%), and a small percentage were admitted for more than four weeks 15 (6%). Sixty four percent of those taking care of the children on the ward were the biological mothers. The other carers included fathers, aunties, grandmothers, uncles, well wishers, stepmothers and brothers among others. The palliative care needs that the children presented with were:

Physical needs: pneumonia 46 (19%), severe acute malnutrition 38 (16%), mild and moderate acute malnutrition 23 (9.6%), respiratory tract infections 22 (9.3%), and tuberculosis 10 (4.2%). Only one child presented with cancer during this period and he had Kaposi sarcoma. The denominator for this section was 243.

Of the 243 children admitted to the ward, 51 (21%) presented with social needs. The social needs included: poor social support 21 (41%), financial instability 16 (31%), and child neglect 4 (8%). The denominator here is 51.

Psychological needs included: ART counselling 127 (36%), HIV counselling and testing for the child and family 63 (18%), adherence support 53 (18%), bereavement support 22 (6%), disclosure of HIV status 7 (2%), supportive counselling for various reasons 14 (4%), and others 11 (3%). The denominator here was 351, which included all counselling diagnoses. It was more than 243 because some of the patients presented with more than one psychological need.

Spiritual needs included: discontinuing ART because of belief in spiritual healing, 18 (81%) loss of hope because of severe ill health, 1 (5%), and others 3 (14%). The denominator here was 20. Only 20 of the 243 children presented with a spiritual need.

## Discussion

The results of the retrospective study indicate that the majority of the children had a diagnosis of HIV and just under half of the sample had received treatment in excess of five years. Key to the findings is the multiple symptoms that the patients were experiencing. Symptoms that require palliative care as recommended by WHO [26]. These results show that palliative care is still essential in the management of these children, i.e. palliative care is necessary even with availability of antiretroviral therapy. It was good to note that the majority (45%) of the children were admitted to the ward for less than one week. A few (6%) were admitted for more than four weeks. These are usually children with complex psychosocial needs or adolescents with poor adherence, such as those failing a second line ART treatment with end stage or terminal HIV conditions like toxoplasmosis, cryptococcal meningitis, extra pulmonary tuberculosis among others; they are admitted for the management of these conditions and end of life care, i.e., in case of some. This is in keeping with previous studies which show that HIV positive children who receive professional care are often hospitalised for many months at the end of life [7].

## Palliative care needs of the HIV exposed and HIV positive children

### Physical needs

The common physical needs identified were pneumonia and severe acute malnutrition. Tuberculosis (TB) and oral candidiasis were also among the opportunistic infections that the children presented with. Pneumonia is a common cause of illness and death among children aged less than five years and those with HIV, particularly the infants are more vulnerable [8]. More than 80% of HIV infected children will develop a respiratory illness sometime during the course of their disease [6]. TB is a major cause of morbidity among HIV infected children [10]. In our study the prevalence was low probably because the majority of the children were on ART and had been in care for a long time. Children are screened for TB at every visit and those found to have it are treated immediately with good outcomes. It is mainly those children who are failing on ART and those who are newly diagnosed with HIV that are frequently diagnosed with TB on the inpatient unit. Severe and moderate malnutrition were some of the other common diagnoses. Other studies have shown that HIV positive children are likely to suffer from severe malnutrition with increased mortality [11]. It is therefore important that such children are managed vigorously according to the national and WHO guidelines. Mildmay Uganda uses these guidelines and has collaboration with the nutrition unit at the national referral hospital. The interventions for the physical needs at the hospital include the appropriate medical treatments such as nutritional rehabilitation, antibiotics for pneumonia, pain relief, and other disease specific treatments. Among all only one child was admitted with cancer during this period. This may also be attributed to the fact that majority of the children were on ART but also Mildmay collaborates with the Uganda Cancer Institute in the care of children with cancer so most of the children would not be admitted at Mildmay. They may only be admitted to Mildmay if only the treatment required is exclusively palliative.

### Social needs

The common social needs were poor social support, financial instability, and child neglect. Good social support is the key to the achievement of proper adherence of ART by children [29]. Financial concerns can be overwhelming for a family with a chronically ill child. Frequent visits to hospitals and care providers for monitoring and treatment are burdensome to families. These demands can either financially bankrupt a family or create such a financial burden that families become vulnerable to a variety of related stressors [12]. It is therefore not surprising that financial instability was one of the issues of the patients in this study. Interventions for social needs at hospital include: working with the family to identify another carer for the child, involvement of local leaders to solve family problems, home visit to assess the situation at home/assess the social support system at home, hired helper brought on board to help take care of the child, legal action, Income Generation Activity (IGA) support, counselling on child care and parenting especially for the young and adolescent parents, placement of children in foster homes where no family member has been identified to take care of the child, or enrollment into hope club (a club for young parents where they are equipped with parenting and income generation or business skills). Mildmay Uganda works with other partners such as the probation office and Uganda Network on Law, Ethics and HIV/AIDS UGANET (a nongovernmental organisation whose mission is to promote human rights based approaches in HIV response to ensure social and legal protection of vulnerable persons) especially for managing the legal aspects of the care of these children. The policies on child protection in Uganda are followed in their management. Reunion with the child’s family or finding a safe place for them to stay is important, as support from one’s family has also been documented as a source of assistance in accessing [13] and adhering to ART [14]. Studies have found financial constraints to be a significant barrier to access and in the adherence to ART [15]. This is why Mildmay Uganda offers IGA support to families with the hope that it will improve their financial status and thus improve their adherence to ART.

### Psychological needs

The common reasons for seeking counselling support from the counsellor as shown in the results section included ART counselling, HIV counselling, and testing the child and family and their adherence to this support. During ART counselling the patients and their families are given information about the HIV and antiretroviral drugs i.e. how HIV is acquired and its effects on the body, how the drugs work, how they should be taken, the need for proper adherence, the expected side effects, and how to detect them among others. The child and family are also assessed for their readiness to start ART. The need for information has been documented by other studies [16, 17]. Mildmay Uganda uses a family centred approach to care so one of the services offered is HIV counselling and testing for the child and family. The need for this type of care has been demonstrated by other studies as well; it is important for the holistic well-being of all family members. Good family-based care is needed for children in Africa to achieve the levels of adherence required for virological suppression [18]. Psychosocial support (PSS) is an essential component of ongoing care for all people living with HIV. PSS is especially critical for children, creating the foundation from which they can establish their identity and place in society, manage their care and live positively, cope with challenges, and plan for their future [19, 20]. Interventions for the psychological needs includes adherence counselling, strengthening the treatment support system for the child, empowerment of the child to take care of their own medications, behavioural change techniques, supportive counselling, pre and post test counselling, and if HIV positive they are enrolled into care, pre and post bereavement support, peer counselling, family meetings and counselling sessions, discordant couple services, disclosure, and last but not least play therapy.

### Spiritual needs

A common spiritual issue was discontinuation of ART because of a belief in spiritual healing. Spirituality is an essential part of the ‘existential domain’ measured in quality-of-life scores. Positive reports on those measures—a meaningful personal existence, fulfillment of life goals, and a feeling that life to that point had been worthwhile—correlated with a good quality of life for patients with advanced disease [21, 22]. Religious convictions may affect health care decision making [23]. Discontinuation of ART is mainly because of a conviction by most that they have been healed by God. Spiritual counselling, which was the major intervention for this need, involves restoring hope in the patients that despite the spiritual healing they need to take ART to keep well. Another study is needed to explore the major reasons for this practice and make evidence based recommendations.

The palliative care needs identified for these children are similar to those for children with cancer especially the need for information and financial and emotional support [24]. In the same regard, a dedicated interdisplinary team is helpful for integrating and coordinating the involvement of the paediatric palliative care clinicians as well as other disciplines e.g. social worker, paediatric psychologists, chaplains/ faith workers etc [25].

## Limitation of the study

This being a retrospective study the data used was not collected for research purposes and may not have been 100% complete.

## Conclusions

The above results show that HIV positive and exposed children plus their families have vast palliative care needs and that a holistic approach is the key in their management. An analytical study to identify or document the factors associated with the above needs is recommended.

## Conflicts of interest

The author(s) declare that they have no conflict of interest.
